# Towards Portable One-Drop Voltammetry with Doped Screen-Printed Electrodes to Control Preservatives: A New Tool for Diuron and Isoproturon in the Paint Industry

**DOI:** 10.3390/s25226987

**Published:** 2025-11-15

**Authors:** Sergio Huertas-Bastidas, Yolanda Moliner-Martínez, Pilar Campíns-Falcó

**Affiliations:** MINTOTA Research Group, Departament de Química Analítica, Facultat de Química, Universitat de Valencia, 46100 Burjassot, Spain; serhuer@alumni.uv.es

**Keywords:** diuron, portable square wave voltammetry, isoproturon, paint industry, iosthiazolinones, water-based paint samples

## Abstract

The use of preservatives such as diuron and isoproturon in the paint industry is essential to protect products against microbial attack. However, these compounds are subject to strict regulation due to the harmful effects they have on the environment and human health. Therefore, analytical strategies to control the production process at paint plants are fundamental to ensure suitable products. In the present work, a low-cost portable square-wave voltammetry device with commercial screen-printed electrodes was proposed to control the starting products and to determine isoproturon and diuron levels in manufactured paint products. Under the optimized conditions (electrolyte HClO_4_ 0.18 M, nickel oxide-doped carbon electrodes, E_SW_ = 0.02 V, E_step_ = 0.0015 V, and ƒ = 15 Hz), the results indicated satisfactory analytical performance, with detection limits of 3.5 and 3.0 mg L^−1^ for isoproturon and diuron, respectively, and precision lower than 7.5% for both biocides. The analytical strategy employed to achieve satisfactory selectivity involved taking advantage of the specific interaction of cysteine with 1,2-benzisothiazol-3(2H)-one (BIT) as a potential interferent in some commercial products and the use of matrix match calibration. A recovery study provided values in the range of 92–104% for accuracy validation. A sample pretreatment step was needed due to the paint composition, and a miniaturized method was proposed here. The novelty of this method lies in the use of a portable voltammetry device in real-world industrial applications to control the paint production process using a cost-effective, time-saving, sustainable, and green protocol. The HEXAGON tool is used for assessing greenness and sustainability. The choice of reagents like HClO_4_ and the minimization of waste from the small volumes used align with the principles of using safer solvents, a key concern in green and sustainable chemistry.

## 1. Introduction

The paint and coating industry is one of the most important markets, with data on Europe noting a consumption of over 40 million tons of paint, amounting to several tens of billions of euros [1]. Water-based paints have displaced solvent-based products, but the addition of preservatives to protect them against microbial attack is necessary. Their use is governed by strict regulations due to the harmful effects of these compounds on the environment and human health [2,3]. Thus, this industry is facing many environmental challenges, with increased focus placed on meeting requirements according to the regulations.

Among the different chemical compounds used as preservatives, triazines, phenylureas, isothiazolinones, and mixtures of these substances are used. Diuron and isoproturon are phenylurea compounds that are widely used as dry film preservatives in coating formulations, particularly for exterior and interior applications, with a film protection effect. Both act primarily as algicidal and secondarily as fungicidal agents, preventing the growth of photosynthetic organisms on the surface of dried coatings. Their presence ensures the long-term esthetic and functional stability of the paint film by inhibiting the biological colonization promoted by humidity and sunlight exposure [4]. When combined, these two compounds provide synergistic performance, extending the duration and range of biocidal protection under varying environmental conditions, such as high humidity, intermittent wetting, or intense solar exposure [5,6].

These preservatives are under the framework of biocidal products: Directive 98/8/EC of the European Parliament, concerning the placing of biocidal products on the market [7] and Commission Regulation (EC) Nº 1451/2007 [7]. These compounds are included in group PT7 as products used for preservation of films or coatings by the control of microbial deterioration in order to protect the initial properties of the surface of materials or objects such as paints, plastics, sealants, wall adhesives, binders, papers, and art works, and are also in group PT6, related to preservatives that are described as products used for the preservation of manufactured products, other than foodstuffs or feeding stuffs, in containers by the control of microbial deterioration to ensure their quality [8]. In addition, their use is limited by Regulation (EU) Nº 528/2012 [9], where the usage limits for individual chemical compounds are listed. Compliance with these regulations is essential for the paint industry, since this mitigates the categorization of products containing these substances as hazardous and avoids their labeling as damaging products. Analytical strategies for developing green and sustainable products are mandatory, and two perspectives can be considered: (i) selective, rapid, and reliable on-site control tools for the paint production process, and (ii) the minimization of the whole analytical process.

Water-based industrial paints are made up of solvents (water and coalescent agents), pigments and fillers, emulsified resins or binders, and additives such as biocides [10]. The analysis of these samples requires a thorough consideration of the matrix dispersion properties. Consequently, it is essential to implement appropriate pre-treatment and quantitation of the commercial paint to ensure accurate results based on the dispersion nature of the matrices. Chromatographic methodologies are commonly used [11,12,13,14,15,16] for the previously mentioned biocides in several matrices, different from paints. In addition, other methodologies such as spectrometry [15], fluorimetry [17,18,19], capillary electrophoresis [20], and electrochemistry [21,22,23,24,25] can be of use.

Electrochemical methods can be attractive due to their fast response, low cost, and selectivity [26]. Moreover, they are suitable for on-site analysis [27] at the plant since portable equipment is already available. Voltammetric measurements can allow multi-component quantification, leading to shorter analysis times. Despite all the benefits described, there are only a few works in the literature concerning the electrochemical determination of the target analytes, and most of them are focused on the use of electrode modifications to optimize their determination in water samples but are not intended for paint industrial processes [28,29,30,31]. Electrochemical techniques are not only powerful for analysis but also for the degradation of persistent pollutants, for example, tetracycline [32], highlighting their dual role in environmental monitoring and remediation.

Square wave voltammetry (SWV) allows faster analysis times compared to other pulse techniques, such as differential pulse voltammetry or normal pulse voltammetry [33]. In addition, its sensitivity is also adequate to determine the target analytes due to the minimal contribution of the non-faradaic currents. Furthermore, the development of advanced nanocomposite materials, such as α-Fe_2_O_3_ nanoparticle-decorated carbon nanotubes [34] or α-zirconium phosphate/carbon nanotube hybrids [35], has significantly enhanced the sensitivity and selectivity of voltammetric sensors. In this work, we leverage the commercial availability and proven electrocatalytic properties of nickel oxide-doped carbon screen-printed electrodes (SPEs) [36] to achieve a balance between performance, cost, and practicality for industrial applications. Nowadays, the use of commercial miniaturized screen-printed electrodes has been proposed for environmental, clinical, and food analysis. However, to the best of our knowledge, no research papers in the domain of the paint industry have been identified.

Hence, the objective and the novelty of the present work were the development of a sustainable and green methodology for portable voltammetric-based analysis from a sample drop using commercial screen-printed electrodes. In addition, a selective determination of diuron and isoproturon, applicable on-site in the paint industrial process, was achieved. The target analytes are limited by European Regulation Nº 528/2012 to 250 mg L^−1^ [9]. 1,2-benzisothiazolin-3-one (BIT) was selected as the target interference, since this isothiazolinone is also used in preservative mixtures added to paint products. The feasibility of monitoring the analytes in starting and paint-manufactured products was demonstrated, with successful results.

Finally, the HEXAGON tool [37,38] was applied for objective comparison of analytical procedures, based on five main criteria: analytical performance, toxicity and safety, residues, carbon footprint, and economic cost. For each criterion, methods are rated on a scale from 0 to 4, represented in a regular hexagonal diagram. HEXAGON aligns with the principles of green and sustainable chemistry, balancing analytical performance with environmental and safety considerations. A lower score indicates a better adaptation of the method to sustainability aspects without compromising analytical reliability. The application of HEXAGON allows for the selection of methodologies that minimize both solvent and energy consumption, optimize performance, and adapt to the specific analytical scenario.

## 2. Materials and Methods

### 2.1. Reagents and Solutions

All reagents used were of analytical grade. Isoproturon (3-(4-isopropylphenyl)-1,1-dimethylurea, Pestanal^®^, >99%), diuron (3-(3,4-dichlorophenyl)-1,1-dimethylurea, Pestanal^®^, ≥98%), BIT (1,2-benzisothiazol-3(2H)-one, Pestanal^®^, >97%), hydrochloric acid, perchloric acid, and cysteine were purchased from Merk (Madrid, Spain). LAMIRSA industrial grade products from Laboratorios Miret S.A. (Tarrasa, Spain): Isoproturon (>98%), diuron (98%), and a solution of BIT (20%) were also used. Ultrapure water was supplied by a Milli-Q system (Millipore (Bedford, MA, USA)). Standards solutions of diuron (101.4 mg L^−1^), isoproturon (101.4 mg L^−1^), and BIT (102.2 mg L^−1^), and their dilutions and mixtures, were prepared by using a real paint liquid phase consisting of a mixture of 63% tap water and 37% TEXANOL^®^ (2,2,4-trimethyl-1,3-pentadiolmonoisobutirat), that was named paint matrix and provided by ISAVAL (Ribarroja del Turia, Spain). Commercial paints were obtained from ISAVAL, too.

### 2.2. Apparatus

Voltammetric measurements were performed with a 910 PSTAT mini-potentiostat (Metrohm International, Herisau, Switzerland), a compact USB-powered instrument connected to a PC. Data acquisition and analysis were performed with PSTAT software version 1.1 (Build 120217). Screen-printed carbon electrodes (SPCEs) and nickel oxide-modified SPCEs (NiO-SPCEs) were supplied by Metrohm Dropsens (Oviedo, Spain). Each strip integrates a three-electrode configuration consisting of a carbon- or NiO-modified working electrode, a silver reference electrode, and a carbon counter electrode, all printed on a ceramic substrate. The electrodes have a circular working area of 4 mm in diameter, and strip dimensions of 3.4 cm × 1.0 cm × 0.05 cm, with silver electrical contacts.

### 2.3. Optimization of the Electrochemical Signal

The voltammetric measurements were performed using the following procedure: 1 mL of the working standard solutions prepared in the paint matrix (target analytes from 10 to 50 mg L^−1^) was mixed with 100 µL of HClO_4_ (2M), and a drop (60 µL) was placed on the screen-printed electrode. All electrochemical measurements were performed at room temperature and in triplicate.

The study of the electrochemical responses, adding cysteine as a modifier, was performed by mixing 600 μL of working standard solutions (up to 50 mg L^−1^) of BIT, isoproturon, and diuron into the paint matrix with 100 µL of HClO_4_ (2M) and 300 μL of aqueous cysteine to achieve a 1 mM concentration. Afterwards, a drop (60 µL) of working standard solution was added to the screen-printed electrodes and measured.

Operational parameters included electrode composition, conditioning time (t_cond_ = 0–400 s), frequency (ƒ_sw_ = 1–20 Hz), pulse amplitude (E_SW_ = 0.005–0.025 V), and potential step (E_step_ = 0.001–0.02 V). 

### 2.4. Analysis of Paint Samples

**Figure 1:**
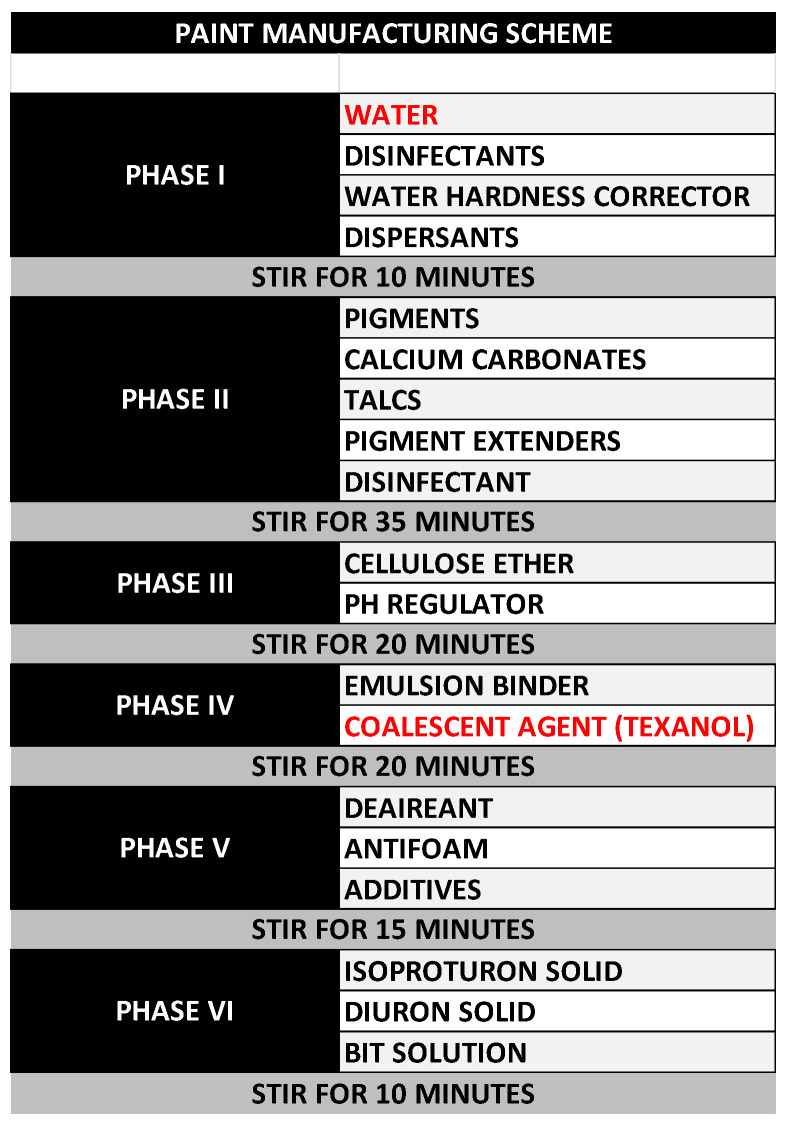
The paint manufacturing scheme used in the factory.

Paint samples were analyzed after a denaturalization and centrifugation process to recover the biocides in the named paint matrix (63% tap water and 37% TEXANOL^®^). The paint manufacturing scheme used in the factory is summarized in Figure 1. At the end of the paint manufacture process, the suspension has a water/TEXANOL proportion corresponding to 63% and 37%, respectively.

First, 1.5 mL of HClO_4_ (2M) was added to 20 mL of paint samples to prepare four replicates, and stirred magnetically for 15 min. Subsequently, 5 mL of the mixture was centrifuged for 45 min at 4500 rpm. Finally, 1 mL of the supernatant was acidified with 100 µL of HClO_4_ (2M). If cysteine was added, 600 μL of the supernatant, 100 µL of HClO_4_, and 300 µL of aqueous cysteine (see Section 2.3) were mixed. Detection was performed by depositing 60 µL of treated samples onto the electrode surface, and measurements were carried out in triplicate using the established conditions.

**Figure 2:**
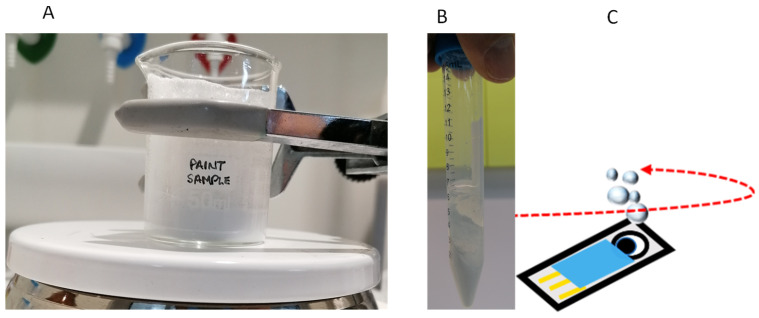
Scheme of the experimental sample treatment. (**A**) Paint sample after acidification with HClO_4_ for denaturalization. (**B**) Paint matrix after centrifugation. (**C**) Screen-printed electrode. For more explanations, see text.

Figure 2A shows the denaturalization process achieved by adding HClO_4_, Figure 2B gives the result of the centrifugation of an aliquot of 5 mL, and Figure 2C displays the electrochemical device that used 60 µL of the mixtures, as indicated in the previous paragraph. Two kinds of paint products (sample 1 and sample 2) were analyzed. Sample 1 contained diuron and isoproturon (labeled concentration of 50 mg L^−1^ each) as preservatives, and sample 2 contained a mixture of diuron, isoproturon, and BIT (labeled concentration 12.5 and 25 mg L^−1^ for diuron and isoproturon, respectively).

## 3. Results and Discussion

### 3.1. Optimization of the Electrochemical Response

The electrochemical responses of diuron and isoproturon are pH-dependent, and the oxidation process favors an acidic pH, since the protonation of amino groups facilitates this process [30,39]. Nickel oxide-doped pure carbon screen-printed electrodes were used due to the presence of nickel oxide and the reduced adsorption of organic molecules, which could provoke an electrode surface saturation and prevent the electric pulse from circulating properly through the electrode [40]. Carbon screen-printed electrodes gave unsatisfactory results.

**Figure 3:**
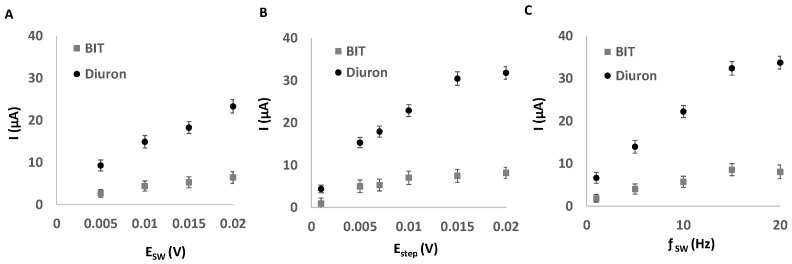
Study of the variation in the peak intensity as a function of (**A**) E_SW_, (**B**) E_step_, and (**C**) ƒ_SW_ for BIT and diuron (25 mg L^−1^).

Instrumental variables such as E_SW_, E_step_, and ƒ, as well as E_cond_ and t_cond_, were studied using diuron. The same study was also performed using BIT as a potential interferent in some paint formulations. Figure 3 shows the variation in the current intensity over E_SW_, E_step_, and ƒ. The results indicated that E_SW_ = 0.02 V, E_step_ = 0.0015 V, and ƒ = 15 Hz (E_cond_ = 0 V and t_cond_ = 0 s) provided satisfactory results in terms of peak shape and intensity for diuron. As can be seen, the variation in the BIT response to changes in instrumental variables was lower than in the case of diuron.

**Figure 4:**
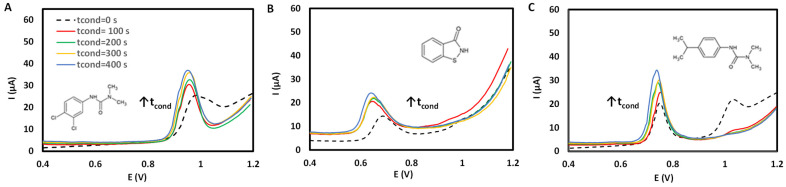
Electrochemical response for (**A**) diuron, (**B**) BIT, and (**C**) isoproturon as a function of conditioning time (tcond/s) (concentration 50 mg L^−1^, electrolyte HClO_4_, E_SW_ = 0.02 V, E_step_ = 0.0015 V, and ƒ= 15 Hz).

Subsequently, t_cond_ was evaluated to equalize the electrodes, and induced changes that facilitated better separation. Figure 4 shows the variation in the voltammogram at different t_cond_ between 0 and 400 s for diuron, BIT, and isoproturon. As can be seen, the variation in t_cond_ improved both the peak shape and the analytical response.

Additionally, the application of cathodic pretreatment was also studied (E_cond_ = −0.2 V). The application of a negative potential in the pretreatment step gave rise to an increase in the analyte response by a factor of 1.5 for the three target analytes. From these results, it can be derived that by employing a t_cond_ = 400 s and the cathodic pretreatment, the peak shape was improved, and the current intensity increased. These conditions were used for further experiments.

**Figure 5:**
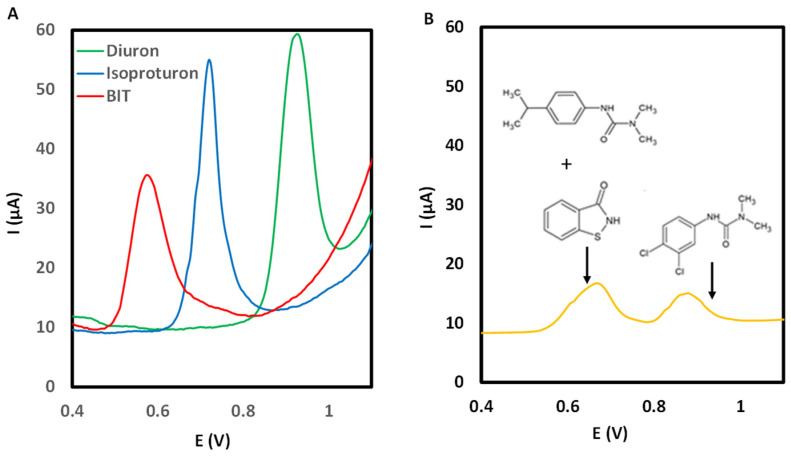
(**A**) Response for individual solution of isoproturon, diuron, and BIT (50 mg L^−1^). (**B**) Voltammogram for a mixture of isoproturon, diuron, and BIT (15 mg L^−1^). Instrumental variables: electrolyte HClO_4_; E_SW_ = 0.02 V; E_step_ = 0.0015 V; ƒ = 15 Hz; t_cond_ = 400 s; and E_cond_ = −0.2 V.

Figure 5A shows the response at a concentration level of 50 mg L^−1^ in the paint matrix (63% tap water and 37% TEXANOL^®^) for individual and mixture of biocides. The oxidation processes were observed at +0.97 and +0.75 V for diuron and isoproturon, respectively. However, the results obtained for a mixture of preservatives showed that for isoproturon and BIT, the resolution was not suitable; indeed, the signals were overlapped (Figure 5B). Under these conditions, only diuron can be properly quantified.

The influence of the supporting electrolyte on the simultaneous determination of the target analytes was studied using HClO_4_ and HCl. The results indicated that HCl did not improve the peak shape nor the intensity, and BIT and isoproturon also presented overlapped peaks. HClO_4_ was maintained for further experiments since the sample pretreatment of paint samples required denaturalization with HClO_4_; therefore, a simplification of the procedure was achieved by using the same reagent as a supporting electrolyte and denaturalization compound.

### 3.2. Study of the Cysteine (Cys) Addition for Increasing Selectivity

**Figure 6:**
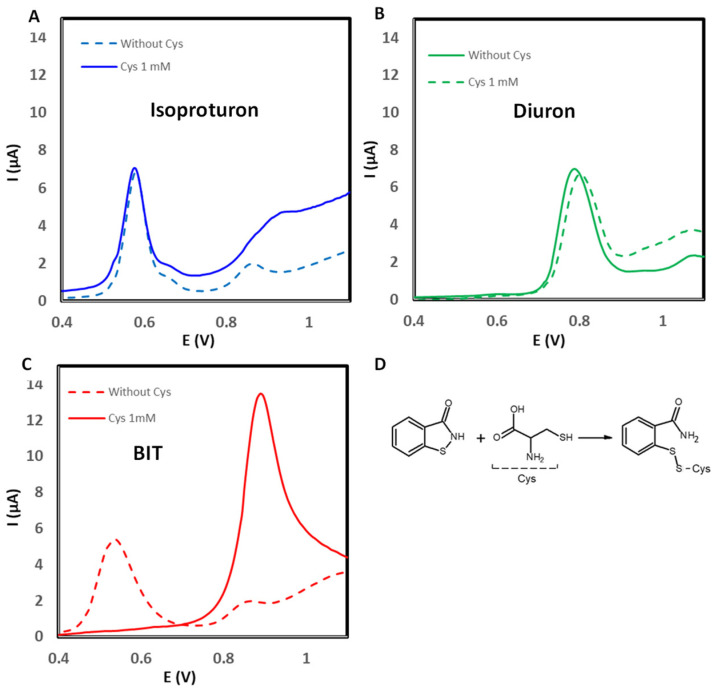
Comparison of the electrochemical response without Cys and adding 1 mM Cys for (**A**) isoproturon, (**B**) diuron, (**C**) BIT, and (**D**) reaction mechanism to form the disulfide compound (concentration level of target analytes and BIT 15 mg L^−1^).

The strategy to selectively determine the phenylureas in paint samples was to take advantage of the interactions of isothiazolinones towards mercapto derivatives [41]. In this study, cysteine was used as a thiol. The electron-deficient sulfur in the N-S bond reacts with nucleophilic groups such as thiols to form disulfide derivatives. The hypothesis was that the formation of the disulfide derivative could induce a variation in the electrochemical response that would allow the selective determination of diuron and isoproturon in preservative mixtures where BIT is also a component. Figure 6 shows the influence of cysteine (1 mM) on the electrochemical response for diuron, isoproturon, and BIT. The addition of cysteine did not modify the response for isoproturon and diuron (Figure 6A,B, respectively). However, in the case of BIT, the electroanalytical response when Cys was added shifted to a higher potential, as shown in Figure 6C. The derivatization of BIT to form the disulfide compound (Figure 6D) modified the potential to 0.9 V. Under these experimental conditions, the addition of Cys allowed for the selective detection of isoproturon; however, the diuron signal was interfered with due to the variation in BIT potential.

### 3.3. Analytical Parameters

The voltammetric responses for target analytes in the paint matrix were evaluated and registered as a function of concentration. Matrix-matched calibration, where the biocides are recovered, was employed. The analytical parameters are summarized in Table 1.

As can be seen in Table 1, there was a good correlation in the working concentration ranges up to 50 mg L^−1^. Quantification levels (LOQs) were 9 and 10 for diuron and isoproturon, respectively. Detection limits (LOD) were calculated from three signal-to-noise ratios. Table 1 shows the values obtained. Taking into account the normal concentration levels of these compounds in the paint samples, LOD and the working intervals met the requirements to determine the target analytes in manufactured products. The method can be employed if the regulated values decrease until the LOQs provided by the developed method. Inter-day precision was also evaluated and expressed as relative standard deviation, RSD. The results shown in Table 1 give RSD values below 7.5%, meaning satisfactory precision for practical industrial application. Several batches (*n* = 5) were assayed, and the achieved precision was lower than 7.5% too.

### 3.4. Analytical Performance in the Paint Industry

Firstly, industrial-grade isoproturon and diuron were assayed in the paint matrix (63% tap water and 37% TEXANOL^®^), and the obtained signals were in accordance with those obtained with analytical grade standards shown in Table 1. The proposed procedure served as a method for quality control for the starting biocides.

Paint samples were analyzed following the procedure described in Section 2.4. A recovery study was conducted in the absence and presence of cysteine. To this aim, paint samples were prepared without biocides in the factory using the procedure indicated in Figure 1 and afterwards, spiked with diuron (12.5 mg L^−1^) and isoproturon (25 mg L^−1^). The acidification process for the denaturalization of the spiked paint base produced an effervescent effect, explained by the reaction between CaCO_3_ present in the paint base and the added acid, producing CO_2_. For this reason, a deaeration process was performed prior to centrifugation. Moreover, the acidification of the sample also promoted separation between the solid and liquid phases in the paint during the centrifugation process (see Figure 2). The high stability of paint binders and additives at basic pH decreased when the pH decreased to acidic values in the denaturalization process. Two phases were obtained that can easily be separated by centrifugation, dragging fillers and colorants to the bottom of the centrifuge tube.

Under these conditions, the response for diuron was evaluated in the absence of Cys, and the response for isoproturon in the presence of Cys. The recovery values were 94 ± 9% and 93 ± 8% for diuron and isoproturon, respectively. These results indicated quantitative extraction and, hence, the procedure was used to determine the target analytes in two kinds of samples as a proof of concept of the proposed methodology. As described in Section 2.4, sample 1 contained diuron and isoproturon (labeled concentration of 50 mg L^−1^ each). Hence, direct analysis was performed for both analytes, since BIT was not an interfering species. However, Sample 2 contained a mixture of diuron, isoproturon, and BIT (labeled concentration 12.5 and 25 mg L^−1^ for diuron and isoproturon, respectively). Then, diuron was directly determined, and isoproturon was determined after the addition of cysteine. Table 2 shows the results obtained for the analyzed samples.

A statistical significance test, Student’s *t*-test, was performed to compare the results obtained with the proposed methodology with the labeled values. The calculated values for *t* were 1.732 and 1.155 for diuron in samples 1 and 2, respectively, and 0.866 and 0.286 for isoproturon in samples 1 and 2, respectively. These results indicated no significant differences with the reference values, taking into account that the critical value for Student’s *t* distribution was 4.303 (95%, df = 3). The results obtained demonstrated satisfactory accuracy and precision, taking into account the objective of the proposed strategy—quality control of diuron and isoproturon in manufactured paint products. Hence, the use of portable square wave voltammetry combined with commercial screen-printed electrodes was demonstrated to be an alternative tool for determining preservatives selectively in paint product matrices by drop sample analysis.

**Figure 7:**
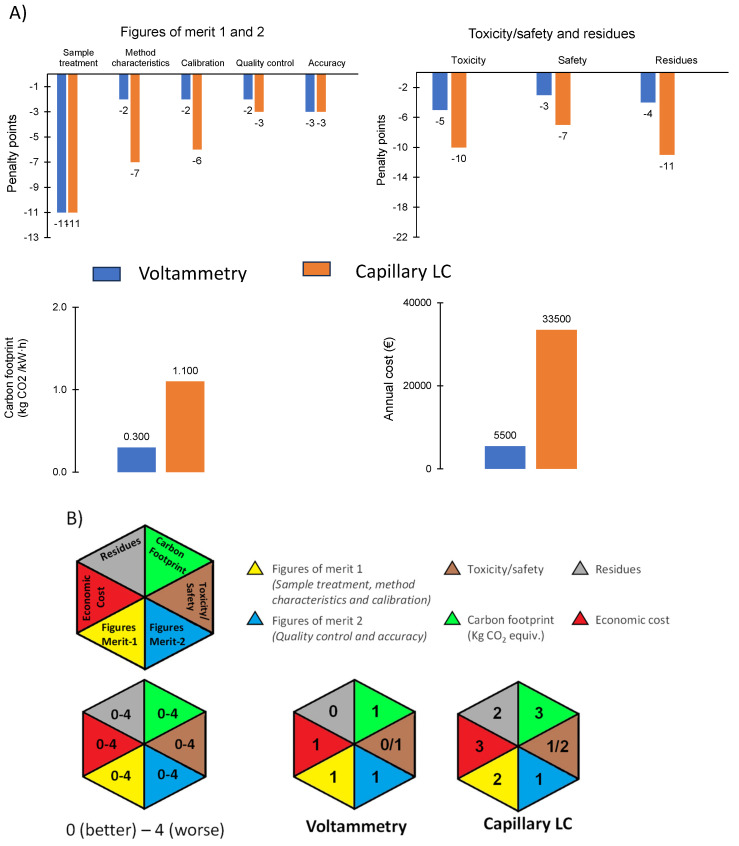
(**A**) Variables considered for establishing HEXAGON tool for voltammetry and capillary LC. **(B**) HEXAGON tool for the two methods.

HEXAGON, as mentioned in Section 1, is in line with the green and sustainable chemistry philosophy, also balancing the figures of merit needed for solving a given problem. Figure 7A shows the penalty points (PP) for the proposed methodology and a procedure based on capillary liquid chromatography (LC) [42], which is a miniaturized technique that improves the greenness and sustainability of conventional LC. The same pretreatment for paint samples proposed here was considered with reference to the LC method, which was developed for water samples.

Other methodologies based on electrochemical measurements or not were not evaluated because, as indicated in Section 1, they were not developed for paint samples. To our knowledge, scientific publications thus far have not been focused on the paint industry. The developed method showed lower penalties in most of the analyzed categories, particularly in terms of toxicity and waste generation. The quantitative carbon footprint calculated from [42,43] and the annual cost (see Figure 7A) highlight its environmentally friendly character. Refs. [37,38,44] give a detailed explanation of how to calculate PPs. A greener alternative for a specific purpose, such as the assessment of paint products in relation to the presence of biocides, is the proposed methodology. As shown in Figure 7B, HEXAGON provides smaller values for the topics tested in the case of voltammetry; except for Figures of merit 2 with a value of 1 in the HEXAGONs for both methods. This allows for the selection of the most appropriate method not only based on analytical performance but also considering environmental impact and practical feasibility. The choice of reagents such as HClO_4_ and the minimization of waste from the small volumes used align with the principles of using safer solvents, a key concern in green and sustainable chemistry.

## 4. Conclusions

In this study, a simple, fast, and cost-effective method for the determination of diuron and isoproturon preservatives in paint matrices was proposed. The determination of isoproturon and diuron in manufactured paint products was successfully performed by commercial, portable voltammetry using nickel oxide-doped carbon electrodes. The use of HClO_4_ as an electrolyte improved the sensitivity of the method. Sample pretreatment was carried out by the acidification of paint samples and a centrifugation step. Afterwards, the studied preservatives can be detected in a single drop of the extract by square wave voltammetry. Selectivity was achieved by taking advantage of cysteine as a modifier. Under these conditions, the target analytes can be resolved from isothiazolinones such as BIT, which can be present together with diuron and isoproturon in some paint formulations. According to these results, this methodology is a promising way to determine the presence of diuron and isoproturon in industrial processes where chromatography is more difficult to use. The proposed strategy enables the determination of preservatives in paints either during or after the manufacturing process, reducing the need for extensive sample pre-treatment and facilitating on-site detection. Its novelty lies in utilizing a commercial portable voltammetry device, offering a cost-effective, time-efficient, green, and sustainable approach for on-site analysis of manufactured paints. The HEXAGON tool indicated that the proposed method is suitable not only based on analytical parameters but also considering greenness and sustainability, as well as practical feasibility.

The drive to achieve such cost-effective and sustainable analytical methods mirrors similar efforts in industrial resource recovery, such as the development of novel separation techniques for valuable materials from waste streams [45], underscoring a unified movement towards greener industrial practices.

## Figures and Tables

**Table 1:** Analytical parameters for the target analytes under the experimental conditions. * Measured concentration of 25 mg L^−1^ for both, isoproturon and diuron.

	Analytical Parameters	
	Isoproturon	Diuron
a ± s_a_	−1.7 ± 1.7	−0.6 ± 1.0
b_1_ ± s_b_ (L mg^−1^)	0.85 ± 0.06	1.00 ± 0.04
R^2^	0.9913	0.9970
LOD (mg L^−1^)	3.5	3.0
* RSD (%), *n* = 10	6.8	7.5

**Table 2:** Found and labeled concentrations in paint samples.

	Labeled Concentration (mg L^−1^)		Found Concentration (mg L^−1^)	
	Isoproturon	Diuron	Isoproturon	Diuron
Sample 1	50.0	50.0	46 ± 4	49 ± 2
Sample 2	25.0	12.5	23 ± 3	13 ± 3

## Data Availability

Data will be provided upon request.
